# Sirtuin1 activator SRT2183 suppresses glioma cell growth involving activation of endoplasmic reticulum stress pathway

**DOI:** 10.1186/s12885-019-5852-5

**Published:** 2019-07-18

**Authors:** Tian Ye, Liwen Wei, Ji Shi, Ke Jiang, Huizhe Xu, Lulu Hu, Lingkai Kong, Ye Zhang, Songshu Meng, Haozhe Piao

**Affiliations:** 1 Department of Neurosurgery, Cancer Hospital of China Medical University, Liaoning Cancer Hospital & Institute, No. 44 Xiaoheyan Road, Dadong District, Shenyang, 110042 Liaoning Province China; 2 Institute of Cancer Stem Cell, Dalian Medical University Cancer Center, 9 Lvshun Road South, Dalian, 116044 China

**Keywords:** Sirt1, Endoplasmic reticulum stress, Glioma, STAT3, NF-κB

## Abstract

**Background:**

Glioblastoma (GBM) is an extremely deadly form of brain cancer with limited treatment options and thus novel therapeutic modalities are necessary. Histone deacetylase inhibitors (HDACi) have demonstrated clinical and preclinical activities against GBM. (Silent mating type information regulation 2 homolog, Sirt1) abbreviated as Sirtuin 1, has been implicated in GBM. We explored the activity of the Sirt1 activator SRT2183 in glioma cell lines in terms of biological response.

**Methods:**

The effects of SRT2183 on glioma cell growth and neurosphere survival were evaluated in vitro using the CCK-8, clonogenic and neurosphere assays, respectively. Glioma cell cycle arrest and apoptosis were determined by flow cytometry. SRT2183-induced autophagy was investigated by detection of GFP-microtubule-associated protein 1 light chain 3 (GFP-LC3) puncta, conversion of the nonlipidated form of LC3 (LC3-I) to the phosphatidylethanolamine-conjugated form (LC3-II). Acetylation of STAT3 and NF-κB in SRT2183-treated glioma cells was examined using immunoprecipitation. The expression levels of anti-apoptotic proteins were assayed by immunoblotting.

**Results:**

SRT2183 suppressed glioma cell growth and destroyed neurospheres in vitro. Furthermore, SRT2183 induced glioma cell cycle arrest and apoptosis, accompanying by upregulation of the pro-apoptotic Bim and downregulation of Bcl-2 and Bcl-xL. Notably, ER stress was triggered in glioma cells upon exposure to SRT2183 while the pre-exposure to 4-PBA, an ER stress inhibitor, significantly antagonized SRT2183-mediated growth inhibition in glioma cells. In addition, SRT2183 induced autophagy in glioma cells and pharmacological modulation of autophagy appeared not to affect SRT2183-inhibited cell growth. Of interest, the acetylation and phosphorylation of p65 NF-κB and STAT3 in glioma cells were differentially affected by SRT2183.

**Conclusions:**

Our data suggest the ER stress pathway is involved in SRT2183-mediated growth inhibition in glioma. Further investigation in vivo is needed to consolidate the data.

## Background

Glioblastoma (GBM) is an extensive and destructive form of neoplastic malignancy, originates from the central nervous system (CNS). The current treatment standard for newly diagnosed GBM comprises surgery, radiation, and chemotherapy, with temozolomide (TMZ). However, GBM cells display inherent resistance to TMZ as well as other cytotoxic drugs. Thus, a crucial and innovative therapeutic treatment is required for effective results for the victims experiencing GBM.

Epigenetic mechanisms, i.e. addition and removal of acetyl groups to the protein possess a critical function with cancer pathogenesis, including GBM. Sirtuin 1, a class III deacetylase relies on NAD (+) has an astonishing capability of deacetylating histones as well as non-histone proteins. Cell propagation, apoptosis, and cellular metabolic activities are concerned with it. Several transcription factors, including TP53, NF-κB/p65, STAT3, and TP53, have been validated as Sirt1substrates [1–3]. Sirt1 is downregulated in GBM tissues and cell lines [4, 5], suggesting a tumor suppressor role of Sirt1 in GBM. However, a recent study demonstrates that neural stem cells need Sirt1 to transform into “neural cancer stem cells” and also aids for the survival of these transmuted cells in a p53 dependent fashion. [6], indicating that Sirt1 functions as an oncogene in GBM.

Pharmacological modulation of cellular acetylation status is being exploited as therapeutic drug targets in GBM [7]. Histone deacetylase (HDAC) blockers namely valproic acid (VPA) and vorinostat reveals experimental and pre-experimental characteristics opposed to GBM [8–12]. SRT2183 was originally described as an activator of Sirt1 [13]. However, several studies demonstrated that SRT2183 do not directly activate Sirt1 [14, 15]. Instead, SRT2183 inhibited p300 histone acetyltransferase (HAT) activity [15], which is known to acetylate many cellular substrates, including TP53 [16]. Another study suggested that SRT2183 exhibited numerous deviant behaviors contrary to cellular catalysts, receptors, conveyor, and ion channels [14]. Nevertheless, Scuto et al. reported that SRT2183 induced growth arrest and the cellular demise of human neoplastic lymphoid cells, affiliated with STAT3 and NF-kB p65 deacetylation [17]. In addition, recent work by Gurt et al. revealed that SRT2183 stimulates AMPK, enhanced Sirt1 expression and reduced RelA/p65 lysine310 acetylation in bone-marrow-derived macrophages [18]. These studies indicate that SRT2183 exerts an antitumor effect. However, whether SRT2183 could exert anti-tumor effects in GBM is unidentified.

In the contemporary investigation, our ambition is to evaluate the effect of evaluated effect SRT2183 in GBM cell lines cultured in vitro. We demonstrate that SRT2183 mediates its antitumor activity at least partly through activation of endoplasmic reticulum stress in glioma cells. These results suggest a potential mechanism by which SRT2183 suppresses glioma cell growth in cultured cells.

## Methods

### Cells, reagents, and plasmid

Glioma cell lines LN229, SF539, SF767, and U87MG were acquired from the American Type Culture Collection (ATCC). Human umbilical vein endothelial cell (HUVEC) was kindly provided by Prof. Pixu Liu (Dalian medical university). LN229 cell line was cultivated in DMEM enriched with 5% fetal bovine serum (FBS).. SF539, SF767, and HUVEC cells were cultured in DMEM with 10% fetal bovine serum (FBS) as an additional nutrient. U87MG cell line requires an MEM medium with 10% fetal bovine serum (FBS) for its nourishment. SRT2183, a specific Sirt1 activator, was purchased from Sellechem and formulated with dimethyl sulfoxide (DMSO) and preserved at -20C. Doxorubicin (DOX) and chloroquine (CQ) were purchased from Sigma. Wortmannin was bought from Calbiochem. Bafilomycin A1 obtained from Millipore.. LY294002 obtained from Cell Signaling Technology (CST). BAY11–7082 was purchased from Medchemexpress. Other reagents were purchased from Selleckchem. GFP-MAP 1LC3B plasmids were purchased from Addgene company.

### Cell viability assay

A number of vital cells were measured through the Cell Counting Kit-8 (CCK8) analysis. Based on highly water-soluble tetrazolium salt by living active cells. 96-well chamber was used to plant LN229, SF539, SF767 U87MG and HUVEC cells respectively (about 6000 cells/well), and were incubated with varying drug (SRT2183) concentrations (0.01, 0.1, 1, 10 and 100 μM) for 24, 48, 72 h.

### Clonogenic assays

LN229, SF539, SF767, and U87MG cells were planted in 6 well chamber (about 1000 cells/well), and then incubated with 10 μM SRT2183. The inverted microscope was used to calculate the replicate clones (comprising 50 or above cells) following 2 weeks.

### Spheroid formation

A mono-layer of both LN229 and U87MG cells were trypsinized and seeded in 96-well ultra-low adherence plates (almost 1000 cells/well) to prepare tumor microspheroids. Basic fibroblast growth factor (bFGF) 10 ng/ml, epidermal growth factor (EGF) 20 ng/ml and 1 × B27 mixed with DMEM/F12 (FBS deprived) medium to cultivate the cells. The reproduced 3D tumor microspheroids were detected and calculated by the inverted microscope following 10 days.

### Analysis of cell cycle arrest and apoptosis by flow cytometry

The cell cycle arrest analysis required the treatment of cells for 24 h with vehicle control and SRT2183 and collected afterward. Likewise, overnight fixation of cells was done in chilled ethanol (70%) at refrigerated temperature. Even more, cells undergo PBS treatment (with 100μg/ml RNase A, 50μg/ml PI, and 0.2% Triton X-100) to assure that DNA was stained solely. Ultimately, the cells were placed at room temperature for incubation and then sorted with the aid of flow cytometer.

For programmed cell death assay, A control vehicle and SRT2183 are required. Then the samples were constrained to the evaluation of membrane reallocation of phosphatidylserine by applying 7-amino-actinomycin-D (7-AAD) and annexin V double staining. Three independent tests were used to examine the fraction of apoptotic cells. Doxorubicin (DOX) was used as a positive control.

### Confocal microscopy

This necessitates the attachment of cells on the round glass coverslips (into the culture plates). Then GFP-LC3 employed to transfect the cells for 24 h. Following the specified procedures, live fluorescent photographs of cells were captured.

### Immunofluorescence staining

Cells were immunostained with primary mouse monoclonal anti-Ki67 antibody (1:200, Cell signaling technology, 9449) and secondary Alexa 488 goat anti-mouse (1:1000, Invitrogen, A-11017) antibody. 5 μg/ml of DAPI (Sigma) dissolved in PBS to stain the cell nucleus. Snapshots were captured with confocal laser microscopy instrument (Leica TCS SP5) using × 60 oil immersion for high-resolution pictures. ImageJ software (for 64 Bit operating system) image podium was used for data interpretation.

### Endogenous immunoprecipitation

Overnight incubation of cell lysates proceeds with NF-κB or STAT3 antibodies (1:200) at 4 °C. Later, washed protein G beads were added and the mixture was incubated at 4 °C for 1 h. Subsequently, the lysis buffer was used to wash the immunoprecipitates for five times, and then the proteins were eluted with the aid of sodium dodecyl sulfate (SDS) sample buffer. Finally, the samples were loaded on SDS polyacrylamide gel electrophoresis (SDS-PAGE) gels and analyzed by western blotting using ac-NF-ΚB (1:1000, Cell signaling technology, 3045S), ac-STAT3 (1:1000, Cell signaling technology, 2523S) antibodies.

### Immunoblot assay

6 cm culture dishes were used to plant LN229 and U87MG, and then the cells were treated with SRT2183. Lysis buffer (Roche, USA) and plastic scrape were used to collect and lyse the cells at 12, 24, and 48 h. Then samples were filled into wells and isolated via 8, 10, and 15% SDS-PAGE. Lastly, the protein bands were shifted to nitrocellulose (NC) membranes (Applygen Technologies Inc. Beijing, China) using a transblot turbo device. Blocking by 5% powder milk dilution in TBST buffer (contains 0.05% Tween 20) for 3 h followed by incubation at 4 °C overnight with primary antibody. The antibodies for CyclinD1 (1:4000, Santa cruz, sc-20,044), P-Rb (Ser780) (1:1000, Cell signaling technology, 9307), Rb (1:1000, Santa Cruz, sc-50), β-actin (110,000, Sigma, A1978), GAPDH (1:5000, Proteintech, 10,494–1-AP), caspase-3 (1:1000, Cell signaling technology, 9662S), PARP (1:1000, Cell signaling technology, 9532S), Bim (1:1000, Cell signaling technology, 2933), Bcl-2 (1:1000, Cell signaling technology, 2870), Bcl-xl (1:1000, Cell signaling technology, 2762), RIP1 (1:1000, Cell signaling technology, 4926), IRE1α (1:1000, Cell signaling technology, 3294), PERK (1:1000, Cell signaling technology, 5683), P-EIF2α (1:1000, Cell signaling technology, 9721), Bip (1:1000, Cell signaling technology, 3177), CHOP (1:500, Cell signaling technology, 2895), LC3 (1:1000, Sigma, L7543), P-Akt (P308) (1:5000, EPITOMICS, 2214–1), P-Akt (P473) (1:1000, Cell signaling technology, 9271), Akt (1:1000, Cell signaling technology, 9272), P-mTOR (1:1000, invitrogen, 44-1125G), mTOR (1:1000, Santa Cruz, sc-8319), P-NF-ΚB p65 (1:1000, Cell signaling technology, 3033), NF-ΚB p65 (1:5000, EPITOMICS, 1546–1), P-STAT3 (Y705) (1:1000, Cell signaling technology, 9138), STAT3 (1:1000, Santa Cruz, sc-482) were used. Recover the primary antibody after overnight, three times membrane washing with TBST. Membranes were then incubated at room temperature with horseradish peroxidase-coupled secondary antibody for 1 h with gentle shaking. Protein blots were then identified by the ECL Western Blot Substrate kit (Thermo Fisher, USA).

### Statistical analysis

One way analysis of variance (ANOVA) was used for the evaluation of data among different groups for each end-point estimated. Various relationships amongst controls and treatments were accessed via Dunnett’s least significant difference (LSD) experiment. The statistically significant value was denoted when *p* < 0.05.

## Results

### SRT2183 suppresses glioma cell growth

**Fig. 1:**
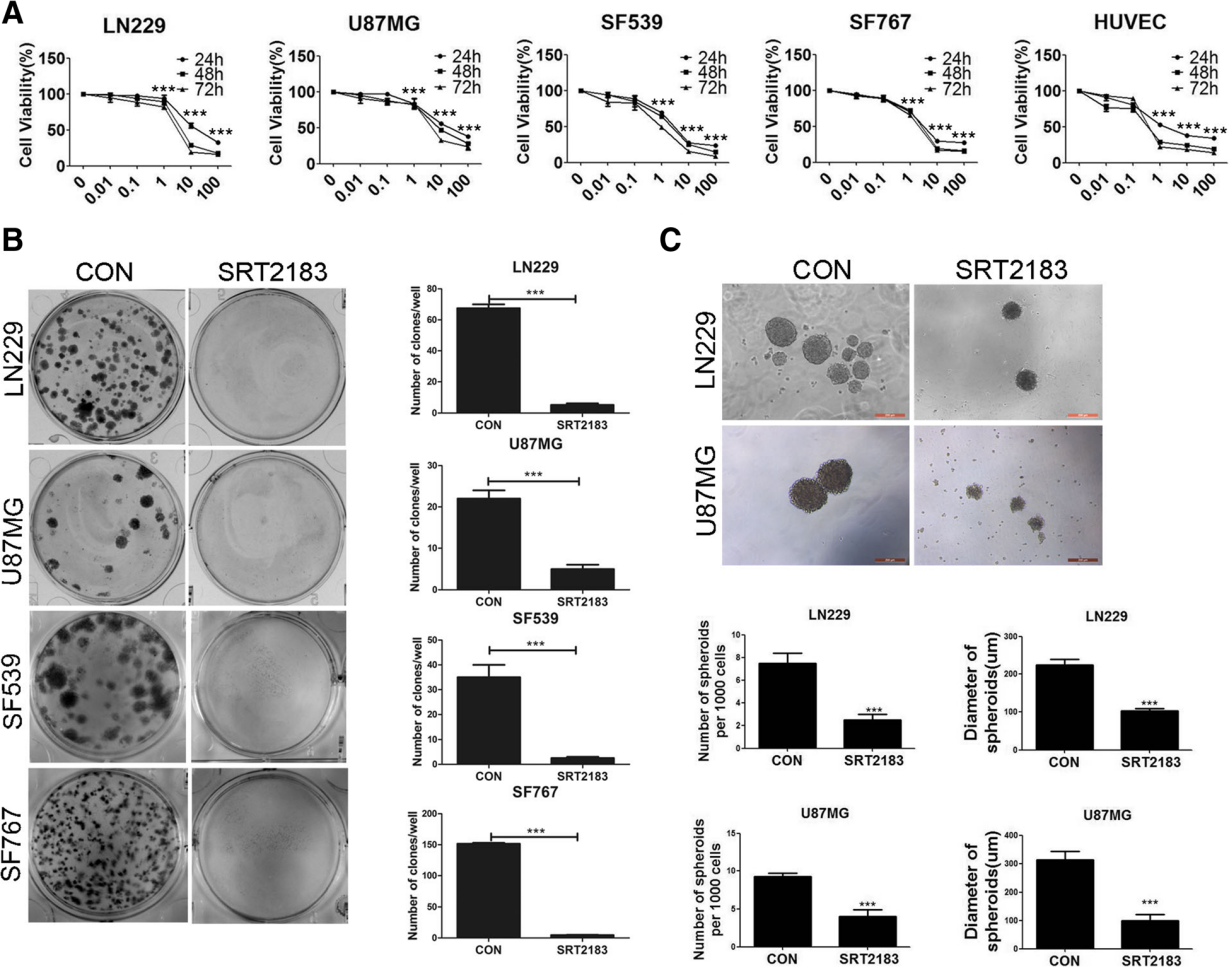
SRT2183 suppresses glioma cell growth and lyses neurospheres in vitro. **a** Modified concentrations of SRT2183 (0.01, 10, 100 μM) were used to treat LN229, SF539, SF767, U87MG, and HUVEC cells with the vehicle for 12, 24 and 48 h. CCK8 analysis displayed the inhibition of cell growth. Results portrayed as the mean ± SEM (***p < 0.001) **b** Cells (LN229, SF539, SF767, and U87MG) undergo treatment with vehicle control and 10 uM SRT2183 and afterward nourished with complete growth medium 2 weeks to test the number of colonies in a clonogenic assay. Results portrayed as the mean ± SEM (***p < 0.001). **c** 3D cultures of LN229 and U87MG cells were treated with vehicle or 10 uM SRT2183 for 10 days and examined for the spheroid formation and spheroid diameter (Scale bar = 200um). Results were declared as a number of microspheroids±SEM (***p < 0.001). The above assays were revised three times with identical results.

We first investigated whether SRT2183 was an effective inhibitor of glioma cell viability. SRT2183 reduced cell viability in glioma cell lines LN229, SF539, SF767, U87MG and HUVEC cell lines in a dose-dependent manner (Fig. 1a), as measured by CCK-8 assay. The half maximal inhibitory concentrations (IC_50_) values at 24 h for the four glioma cell lines measured ranged from 4 μM to 24.5 μM. Viability was reduced by 50% at 5 μM of SRT2183 in the four glioma cell lines tested. SRT2183 at a concentration of 10 μM was used throughout the study. Clonogenic assays showed that SRT2183 decreased significantly the capability of cells to grow clonally after a 2-week treatment (Fig. 1b). Latterly, the LN2299 and U87MG microspheroids were substantially lessened in number and volume after 10 days treatment with SRT2183 (Fig. 1c).

### SRT2183 induces glioma cell cycle arrest and apoptosis

**Fig. 2:**
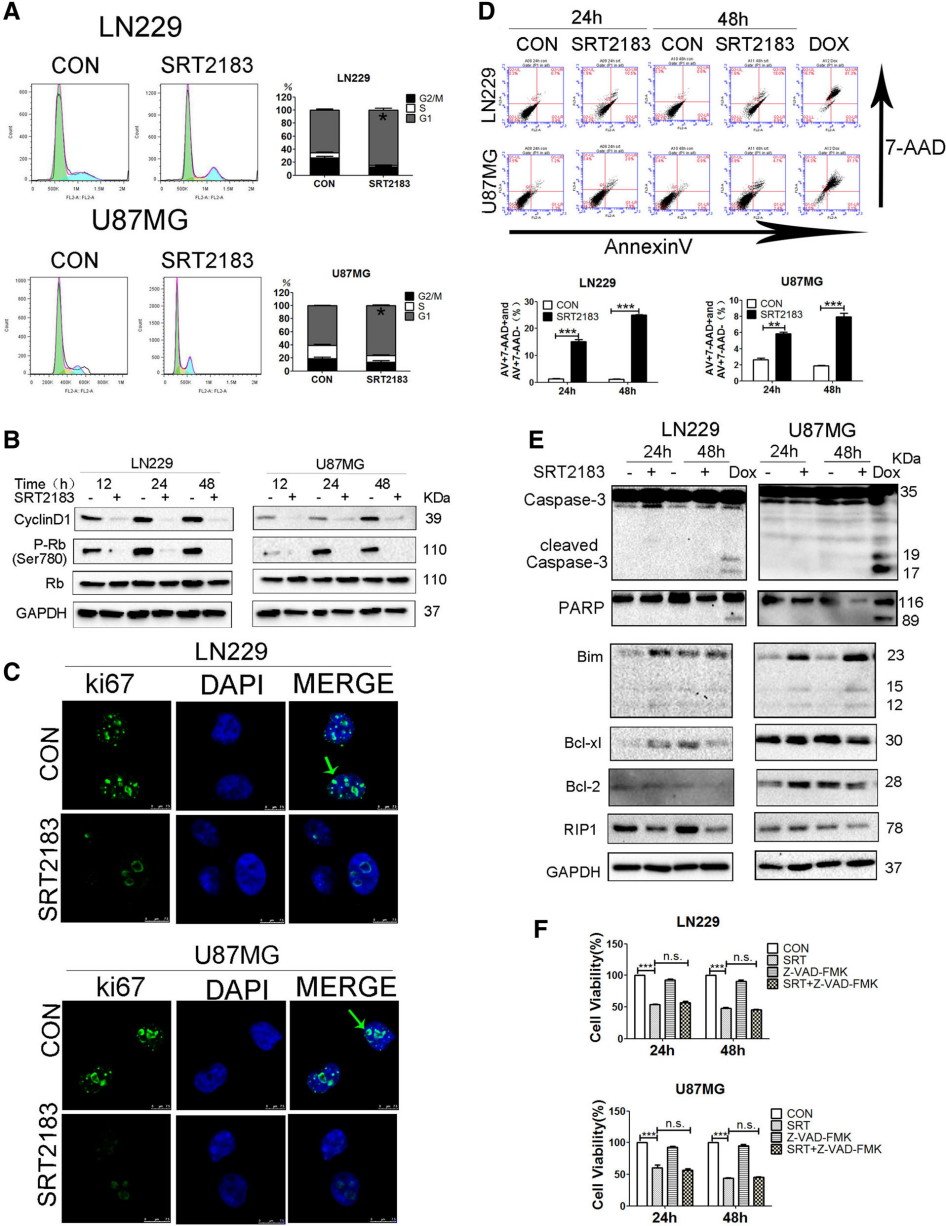
SRT2183 induces glioma cell cycle arrest and apoptosis. **a** LN229 and U87MG cells were treated with vehicle or 10 μM SRT2183, the cells were stained with PI for cell cycle analysis after 24 h. Results portrayed as the mean ± SEM (***p < 0.001). and were analyzed by FACS after staining with propidium iodide for cell cycle analysis at 24 h. Results represent as the mean ± SEM (*p < 0.05). **b** LN229 and U87MG cells were treated with vehicle or 10 μM SRT2183 for 12, 24, 48 h, protein levels of CyclinD1, P-Rb, Rb, and GAPDH were analyzed by immunoblot (IB) (upper panel). LN229 and U87MG cells were treated with vehicle or 10 μM SRT2183 for 24 h. Location of ki67 identified by fluorescent immunostaining. Nucleus stained with DAPI (lower panel). **c** LN229 and U87MG cells were treated with vehicle or 10 μM SRT2183, the cells were assessed by immunofluorescence staining with ki67 and DAPI **d** LN229 and U87MG cells were treated with vehicle or 10 uM SRT2183 for 24 and 48 h. AnnexinV/7-AAD double-staining was using for apoptosis analysis by FACS, 5 uM concentration of Doxorubicin was taken as a positive control. **e** LN229 and U87MG cells were treated for 24 and 48 h using SRT2183 and expression of Caspase-3, PARP, Bim, Bcl-xl and Bcl-2 were examined by IB. 5 uM concentration of Doxorubicin were taken as a positive control. **f** LN229 and U87MG cells pre-treated with Z-VAD-FMK (50 uM) then with the vehicle control or 10 μM SRT2183 after 24 and 48 h, the number of viable cells were quantified by CCK-8 assay. The above experiments were performed three times, (*0.01 < *p* < 0.05, **0.001 < *p* < 0.01, ***p < 0.001, n.s. = not significant).

To investgate the mechanism by which SRT2183 inhibits glioma cell proliferation, we need to confirm either SRT2183 influenced the mitotic cycle by flow cytometry.. For this, we witnessed that the percentage of LN229, U87MG raised by SRT2183 at the G1 phase of the cell growth cycle. Cells show a concurrent decline in G2 and S phase. So suggesting about G1 phase halt. SRT2183 promoted a decrease of LN299 and U87MG cells in S phase and an increase of cells in G2/M (Fig. 2a). Coherent exposition to SRT2183 provoked a prominent decrease in G1 phase-related protein such as Cyclin D1 and pRb in LN229 and U87MG cell lines (Fig. 2b, upper panel), accompanied by a decrease in ki67 staining in SRT2183-treated glioma cells (Fig. 2b, lower panel). Based on these observations, we next checked whether SRT2183 induced apoptosis in glioma cells, using Annexin V-7-amino-actinomycin-D (7-AAD) double staining. Doxorubicin (DOX) was used to monitor cell death. Data presented in Fig. 2c show that SRT2183 fostered Annexin V- percentage and position cell number in 7-AAD-negative and a 7-AAD-positive cell population, indicating that SRT2183 promotes both apoptotic and other forms of cell death in glioma cell lines. To further assess whether SRT2183 could trigger apoptosis in glioma cells we determined the activation of the apoptotic pathway by immunoblot assay. No significant change in caspase-3 processing and Poly (ADP-ribose) polymerase (PARP) cleavage, two classical apoptosis markers, was observed in LN299 and U87MG cells following treatment with SRT2183 (Fig. 2d). As expected, DOX treatment induced evident cleavage of caspase-3 and PARP in these cells (Fig. 2d). Interestingly, the protein levels of RIP1, which serves as a necroptosis marker, were downregulated in SRT2183-treated glioma cells (Fig. 2d).

To investigate the mechanism of apoptosis further, we checked the expression levels of pro- and anti-apoptotic proteins after SRT2183 treatment in LN229 and U87MG cells. We observed that the expression levels of the pro-apoptotic protein, Bim, was upregulated in SRT2183-treated glioma cells (Fig. 2d). An obvious decrease in the expression levels of the pro-survival proteins Bcl-xL and Bcl-2 was detected in either LN229 or U87MG cells following a 48 h treatment with SRT2183 (Fig. 2d). Notably, pretreatment with Z-VAD-FMK, a pan-caspase inhibitor, could not block SRT2183-mediated inhibitory effects on glioma cell growth (Fig. 2e). Together, these data suggest that SRT2183 might induce caspase-independent apoptosis and that a small portion of glioma cells might underwent apoptosis upon SRT2183 treatment. Thus it could not be excluded that SRT2183 might elicit other forms of cell death in glioma cells.

### ER stress plays a role in SRT2183-induced glioma cell death

**Fig. 3:**
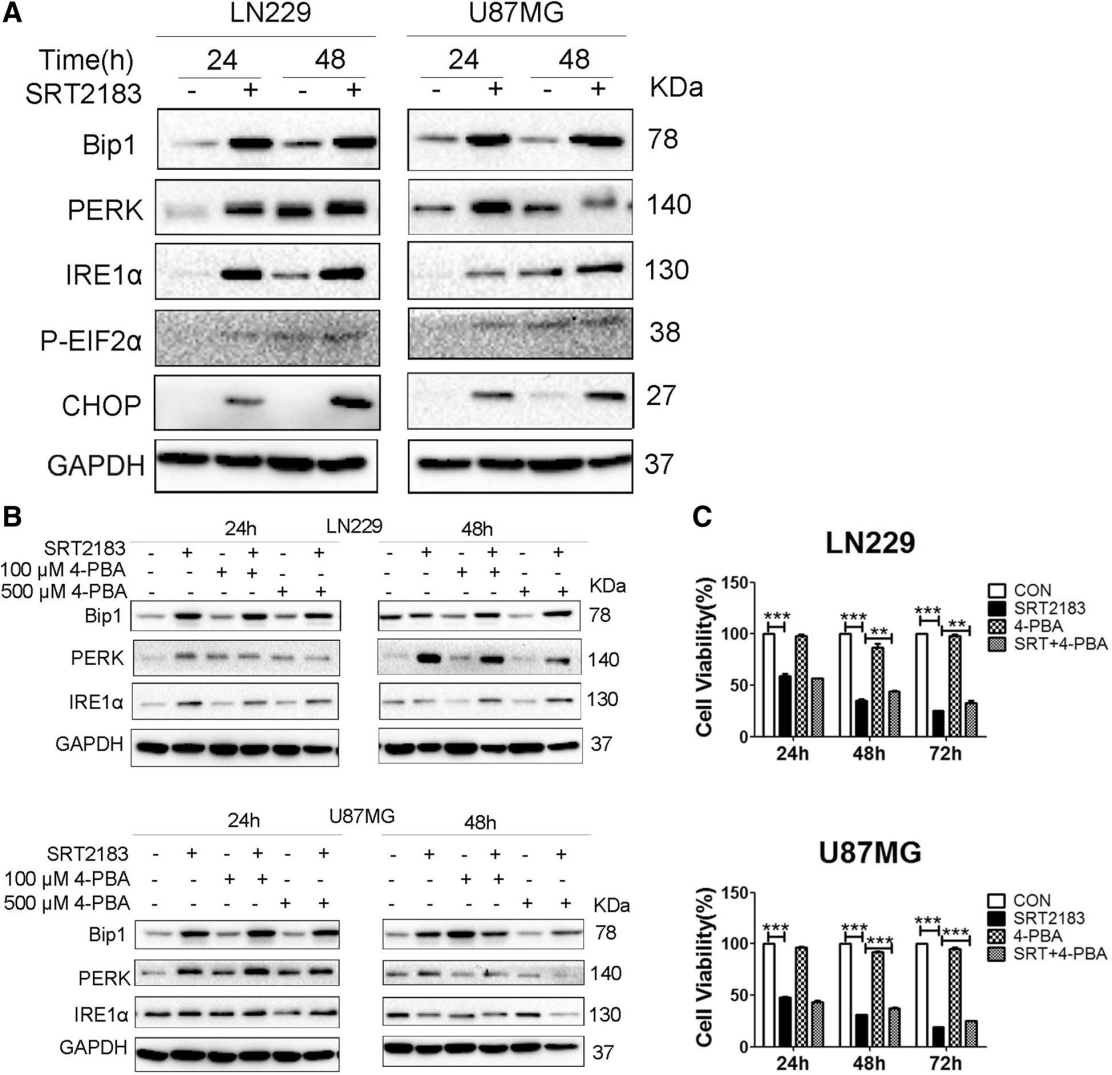
**a** ER stress plays a role in SRT2183 promoted glioma cell death. LN229 and U87MG cells were treated with vehicle or 10 μM SRT2183 for 24, 48 h, expression levels of Bip1, PERK, IRE1α, P-EIF2α, and CHOP were analyzed by IB. **b** LN229 and U87MG cells were pre-treated with vehicle, 4-PBA (100 μM and 500 μM), and then the cells were treated with vehicle or 10 μM SRT2183 for 24, 48 h, after collected the cell lysate, expression levels of Bip1, PERK, and IRE1α were measured by IB. **c** LN229 and U87MG cells were pre-treated with 4-PBA (500 μM), and then the cells were treated with vehicle or 10 μM SRT2183 for 24, 48, 72 h, cell proliferation was measured by CCK-8 assay. The above experiments were performed three times with comparable results. Results portrayed as the mean ± SEM (**0.001 < *p* < 0.01, ***p < 0.001).

We next examined whether other forms of cell death such as ER stress were involved in SRT2183-induced growth inhibition. As illustrated in Fig. 3a, the expression levels of several classical ER stress signaling markers including Bip, IRE1α, PERK and phosphorylation of p-eIF2α, were robustly increased in SRT2183-treated LN229 and U87MG cells at 24 and 48 h postexposure, indicating induction of ER stress pathway. In addition, we observed robust expression of CHOP, a protein involved in ER stress-induced cell death, in SRT2183-treated glioma cell lines (Fig. 3a). Importantly, pretreatment with an ER stress inhibitor (4-Phenylbutyric acid, 4-PBA), dose-dependently blunted SRT2183-induced increase of the protein levels of PERK in both LN229 and U87MG cells (Fig. 3b). Furthermore, pre-exposure to 4-PBA significantly antagonized SRT2183-mediated growth inhibition in LN229 and U87MG cells (Fig. 3c), indicating the role of ER stress in SRT2183-induced glioma cell death.

### SRT2183 induces autophagy in glioma cells

**Fig. 4:**
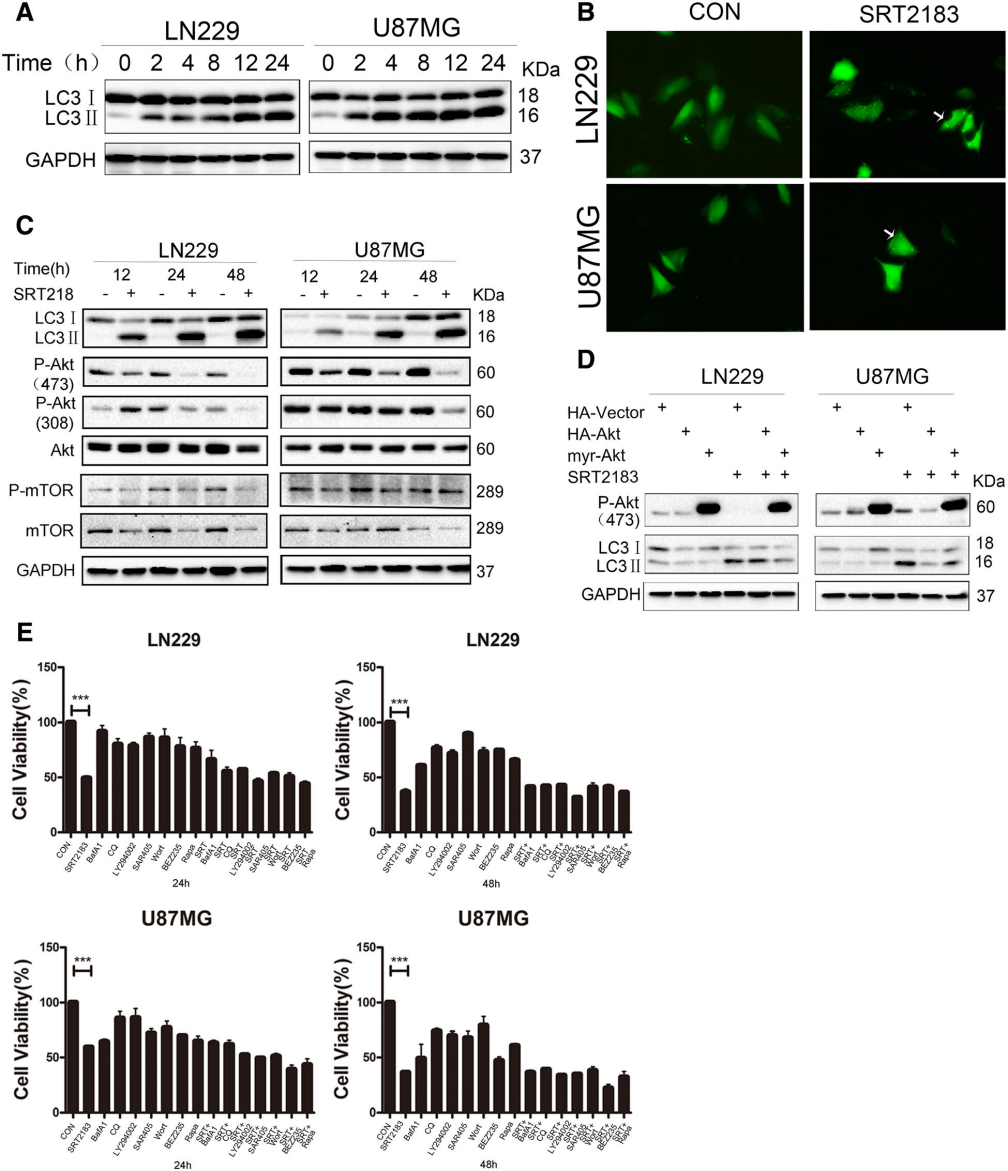
**a** SRT2183 induces autophagy in glioma cells. LN229 and U87MG cells were treated with vehicle or 10 μM SRT2183 for 2, 4, 8, 12, 24 h, expression levels of LC3 and GAPDH were measured by IB analysis. **b** LN229 and U87MG cells were pre-transfected with GFP-LC3,after 24 h the cells were treated with vehicle or 10 uM SRT2183 for 24 h. The results were observed by confocal microscope. **c** LN229 and U87MG cells were treated with vehicle or SRT2183 for 12, 24 and 48 h. Expression levels of LC3, P-Akt (473), P-Akt (308), Akt, P-mTOR, and mTOR were analyzed by IB. GAPDH was used as a control for equal loading. **d** LN229 and U87MG cells were transfected with HA-Vector, HA-Akt, and myr-Akt for 24 h, then they were treated with vehicle or SRT2183 for 24 h. Expression levels of P-Akt (473) and LC3 were analyzed by IB. **e** LN229 and U87MG cells were treated with vehicle or SRT2183 following pre-treatment with either autophagy inducers BEZ235 and Rapamycin (Rapa), or autophagy inhibitors Bafilomycin A1 (BafA1), Chloroquine (CQ), and SAR405, or PI3K inhibitors LY294002 and Wortmannin (Wort). CCK8 analysis was employed to determine cell growth inhibition. The above experiments were performed three times with comparable results. Results portrayed as the mean ± SEM (***p < 0.001).

Stimulation of autophagy is directly related to the triggering of the ER stress pathway [19]. Given that, ER stress in glioblastoma is caused by SRT2183, so we wanted to seek the function of SRT2183 treatment with the activation of autophagic mechanisms. Various time checks were set after the treatment of LN229 and U87MG with SRT2183, and then immunoblotting was used for the detection of MAP 1LC3B-II (i.e. LC3II), the classic autophagy indicator. Figure 4a represents, SRT2183 initiate substantial LC3 modification in glioblastoma cells (from cytosol localized LC3I to lipidated, autophagosome-strapped LC3II) in a certain time period. For the verification of enhanced LC3II, exposure of glioma cells with SRT2183 have some relation with the autophagosomal construction. So we investigate it by MAP 1LC3B puncta. Transfection of LN229 cells with an overexpressed GFP-MAP 1LC3B plasmid with a control GFP-expressing vector, then add the plasmid suspension to the cells for 24 h for the consequent detection of green fluorescent puncta of MAP 1LC3B via immunofluorescent imaging technique. Then comparison with control cells revealed about significant improvement in the establishment of exogenous GFP/LC3 puncta in LN229 and U87MG cells (Fig. 4b), by affirmation of autophagosomal development by SRT2183 in overexpressed GFP-MAP 1LC3B glioblastoma cells. Analogous results were gathered after SRT2183 treatment on U87MG cells (data not shown). In an exploration of the mechanism of SRT2183-dependent autophagic cell death in glioma cell lines, we found the AKT/mTOR pathway phosphorylation, which has negative regulatory effects on autophagy [20]. ate of phosphorylation of AKT becomes downregulated at serine 473, pAKT-S473, denoted in SRT2183 infected LN229 and U87MG cells (Fig. 4c). Decreased phosphorylation of AKT at theorine 308, pAKT-T308, was observed in LN229 cells after a 24 h SRT2183 treatment and in U87MG cells at 48 h postexposure (Fig. 4c). In addition, SRT2183 exposure decreased the phosphorylation levels of mTOR in both glioma cells at 12 and 24 h postexposure (Fig. 4c). Of note, a decrease in the total protein levels of mTOR was detected in both glioma cells exposed to SRT2183 (Fig. 4c). These data indicate that the AKT/mTOR pathway might contribute to SRT2183-triggered induction of autophagy in glioma cells. To study further the role of AKT in SRT2183-induced autophagy in glioma cells, we transfected the glioma cells with either wild type AKT or myr-AKT (activated AKT) or vector in LN229 and U87MG cells, followed by exposure of the cells to SRT2183 for 6 h and subsequent examination of LC3II abundance by immunoblotting. As shown in Fig. 4d, myr-AKT profoundly inhibited SRT2183-triggered increase of the LC3II levels in both cell lines.

We next asked whether modulation of autophagy would interfere with SRT2183-mediated downregulation of glioma cell viability. To this aim, LN229 and U87MG cells were treated with either autophagy inducers BEZ235 and Rapamycin, or autophagy inhibitors BafA1, CQ and SAR405, or PI3K inhibitors LY294002 and Wortmannin, in combination with SRT2183 or alone for varying time points. The beneficial concentrations each mixture from these amalgamates were chosen by a dose-responsive experiment to avoid cytotoxic elements (data not shown). Figure 4e shows that compared to cells exposed to STRT2183 alone, all the tested pharmacological modulators did not significantly affect SRT2183-induced cell death in LN229 and U87MG cells at all the observed time points.

### Pharmacological inhibition of either NF-κB or STAT3 enhances SRT2183-mediated glioma cell death

**Fig. 5:**
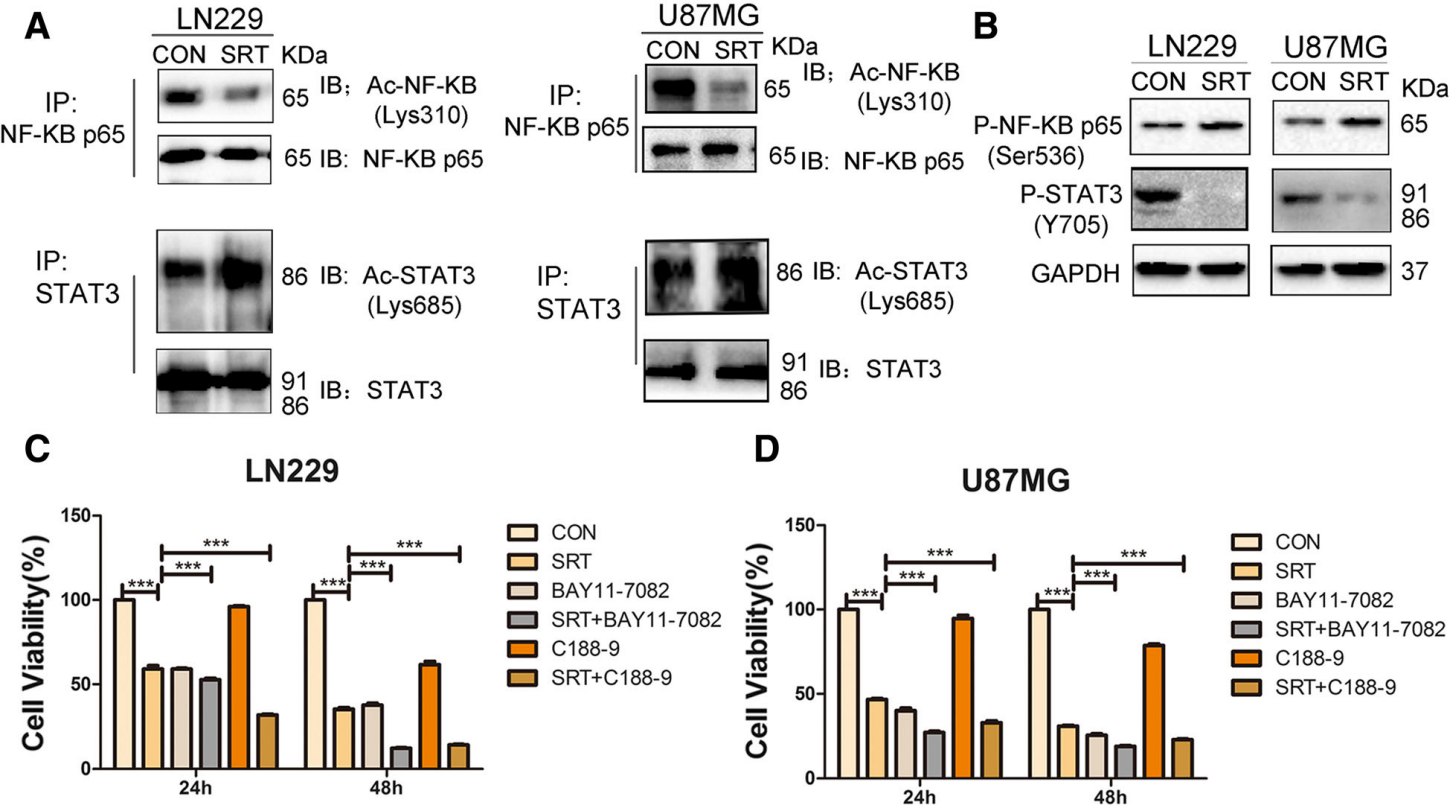
Pharmacological inhibition of either NF-κB or STAT3 enhances SRT2183-mediated glioma cell death. **a** lysates from LN229 and U87MG cells were collected to analyze acetyl-STAT3 and acetyl-NF-κB expression levels by Immunoprecipitation (IP) and IB. The expression levels of total STAT3 and total NF-κB p65 were measured as a loading control. Three independent experiments were used to illustrate the results. **b** LN229 and U87MG cells were treated with vehicle or 10 μM SRT2183 for 24 h, protein levels of P-NF-κB p65 (Ser536) and P-STAT3 (Y705) were analyzed by IB. LN229 (**c**) and U87MG (**d**) cells were treated with vehicle or SRT2183 following pre-treatment with NF-κB inhibitor BAY11–7082 or STAT3 inhibitor C188–9, CCK8 analysis was employed to determine cell growth inhibition. The above experiments were performed three times with comparable results. Results portrayed as the mean ± SEM (***p < 0.001).

Given the inhibitory effects by SRT2183 on glioma cell growth, we examined whether SRT2183 affects the acetylation of STAT3 and NF-κB, two well-known substrates of Sirt1 in glioma cells [1, 2]. As shown in Fig. 5a, SRT2183 exposure for 24 h decreased the acetylation of p65 NF-κB at Lys-310 in LN229 and U87MG cells while the acetylation of STAT3 at Lys-685 was not affected in the two cell lines. In addition, tyrosine phosphorylation levels of STAT3 (pSTAT3Y705) were remarkably decreased in both LN229 and U87MG cells upon a 24 h exposure to SRT2183 while phosphorylation levels of p65 remain largely unchanged (Fig. 5b).

We next investigated whether targeting of NF-κB or STAT3 would exert an effect on SRT2183-induced glioma cell death. Figure 5c shows that both NF-κB inhibitor BAY11–7082 and STAT3 inhibitor significantly enhanced SRT2183-mediated cell death in LN229 cells. Similar results were observed in U87MG cells treated as in LN229 cells (Fig. 5d).

## Discussion

In the present study, we examined the function and characteristics of SRT2183 (a Sirt1 stimulator) in several glioblastoma cells. SRT2183 induces growth arrest and apoptosis. These biological effects were at least partly associated with increased ER stress triggered by SRT2183 in glioma cells. Therefore, our data suggest that the ER stress pathway is involved in SRT2183-mediated growth inhibition in glioma.

SRT2183 was originally documented as an activator of Sirt1 for the treatment of type- 2 diabetes [13]. A recent study showed that SRT2183 inhibit the RANKL stimulated osteoclast formation in bone marrow-stemmed macrophages. [18]. The antitumor implications of the SRT2183 were unexplored. Before investigated by Scuto et al. verified about deacetylation of STAT3 and NF-kB p65 associated with SRT2183 or another Sirt1 stimulatorand SRT501, these two inhibitors both induce apoptosis in human malignant lymphoid cells and activate the expression of DNA damage response genes [17]. Consistently, we observed SRT2183 suppresses glioma cell growth both in 2D and in 3D cultural conditions, indicating that SRT2183 might display antitumor effects in a broad type of cancers. Similar to the investigation in malignant lymphoid cells [17], we observed that SRT2183 also halts the mitotic cycle and cellular demise in glioma cell lines accompanied by an increase of Bim and decrease of Bcl-2 and Bcl-xL. In addition, we showed that the growth inhibition of glioma cells by SRT2183 was partly due to an increased ER stress as the ER stress inhibitor 4-PBA significantly antagonized the effects of SRT2183. Of note, although SRT2183-induced ER stress was accompanied by increased autophagy, targeting autophagy with various autophagy modulators did not significantly impact on the growth inhibitory effects of SRT2183 on glioma cells, suggesting that autophagy might not be involved in SRT2183-induced growth inhibition of glioma cells. Taken together, the main effect of SRT2183 in glioma cells is to induce ER stress-dependent growth inhibition.

It is well established that NF-κB and STAT3 are Sirt1 substrates. Acetylation of p65 NF-κB at Lys-310 and STAT3 at Lys-685 are critical for their transcriptional activity respectively [2, 21, 22]. Giving that Sirt1 represses NF-κB and STAT3 activities by deacetylating p65 NF-κB at Lys-310 and STAT3 at Lys-685, Scuto et al. demonstrated that SRT3183 induces deacetylation of STAT3 and NF-κB p65 malignant lymphoid cell lines while both total and phosphorylation of STAT3 levels are repressed and phosphorylation of NF-κB p65 is not affected [17, 18]. In our study, the acetylation of p65 NFκB at Lys-310 in glioma cells was declined by SRT2183 while the phosphorylation levels of p65 NF-κB not affected. Of interest, we observed a profound decrease in the phosphorylation levels of STAT3 in SRT2183-treated glioma cells while the acetylation of STAT3 at Lys-685 was not significantly affected. Thus, SRT2183 might exert a tumor or cell-specific effects on the activation of Sirt1 substrates. Nevertheless, the combination of SRT2183 with either an NF-κB inhibitor or a STAT3 inhibitor had greater anti-tumor effects in glioma cells compared to SRT2183 alone.

It should be pointed out that although SRT2183 was originally documented as a Sirt1 activator [13], a study by Pacholec et al. revealed that Sirt1 is not promptly triggered by SRT2183 [14]. Of interest, Gurt et al. found that SRT2183 could activate Sirt1 in osteoclasts. Anyhow in different circumstances, Sirt3 can be disturbed by the scarcity of Sirt1 [18], indicating that SRT2183 might have other targets in addition to Sirt1. Indeed, SRT2183 was shown to activate AMPK in osteoclasts [18]. In this study, we observed that SRT2183 downregulates the phosphorylation levels of AKT as well as mTOR in the tested glioma cells. Of interest, it has been well known that either activation of AMPK or inactivation of AKT/mTOR promotes autophagy. Therefore, in addition to AKT/mTOR, AMPK might also contribute to SRT2183-induced autophagy in glioma cells.

## Conclusions

In the current study, we present evidence that the Sirt1 activator indicates growth restrictive and pro-apoptotic activity in glioma cells. At least one of the mechanisms of action is mediated through increased ER stress, which might be the consequences of STAT3/NF-κB inhibition. Given the growth inhibitory effects by SRT2183 on glioma cells in vitro, it will be of translational significance to explore the in vivo anti-glioma effects of SRT2183 in the future study.

## Data Availability

All relevant data are within the paper. In addition, the raw data for Figs. 1, 2, 3, 4 and 5 have been uploaded to Figshare (10.6084/m9.figshare.7967312).

## Abbreviations

3D: Three-dimensional
4-PBA: 4-Phenylbutyric acid
7-AAD: 7-amino-actinomycin-D
CCK-8: Cell Counting Kit-8
CQ: Chloroquine
ER: Endoplasmic reticulum
FBS: Fetal bovine serum
GBM: Glioblastoma
GFP: Green fluorescent protein
IC_50_: The half maximal inhibitory concentrations
LC3: Microtubule-associated protein 1 light chain 3
mTOR: Mammalian target of rapamycin
PARP: Poly (ADP-ribose) polymerase
PERK: Protein kinase-like endoplasmic reticulum kinase

## References

[1] Yeung F, Hoberg JE, Ramsey CS, Keller MD, Jones DR, Frye RA, Mayo MW (2004). Modulation of NF-kappaB-dependent transcription and cell survival by the SIRT1 deacetylase. EMBO J.

[2] Nie Y, Erion DM, Yuan Z, Dietrich M, Shulman GI, Horvath TL, Gao Q (2009). STAT3 inhibition of gluconeogenesis is downregulated by SirT1. Nat Cell Biol.

[3] Chalkiadaki A, Guarente L (2012). Sirtuins mediate mammalian metabolic responses to nutrient availability. Nat Rev Endocrinol.

[4] Lages E, Guttin A, El Atifi M, Ramus C, Ipas H, Dupre I, Rolland D, Salon C, Godfraind C, deFraipont F (2011). MicroRNA and target protein patterns reveal physiopathological features of glioma subtypes. PLoS One.

[5] Romeo SG, Conti A, Polito F, Tomasello C, Barresi V, La Torre D, Cucinotta M, Angileri FF, Bartolotta M, Di Giorgio RM (2016). miRNA regulation of Sirtuin-1 expression in human astrocytoma. Oncol Lett.

[6] Lee JS, Park JR, Kwon OS, Lee TH, Nakano I, Miyoshi H, Chun KH, Park MJ, Lee HJ, Kim SU (2015). SIRT1 is required for oncogenic transformation of neural stem cells and for the survival of “cancer cells with neural stemness” in a p53-dependent manner. Neuro-Oncology.

[7] Galanis E, Anderson SK, Lafky JM, Uhm JH, Giannini C, Kumar SK, Kimlinger TK, Northfelt DW, Flynn PJ, Jaeckle KA (2013). Phase II study of bevacizumab in combination with sorafenib in recurrent glioblastoma (N0776): a north central cancer treatment group trial. Clin Cancer Res.

[8] Krauze AV, Myrehaug SD, Chang MG, Holdford DJ, Smith S, Shih J, Tofilon PJ, Fine HA, Camphausen K (2015). A phase 2 study of concurrent radiation therapy, Temozolomide, and the histone deacetylase inhibitor Valproic acid for patients with glioblastoma. Int J Radiat Oncol Biol Phys.

[9] Galanis E, Jaeckle KA, Maurer MJ, Reid JM, Ames MM, Hardwick JS, Reilly JF, Loboda A, Nebozhyn M, Fantin VR (2009). Phase II trial of vorinostat in recurrent glioblastoma multiforme: a north central cancer treatment group study. J Clin Oncol.

[10] Chinnaiyan P, Chowdhary S, Potthast L, Prabhu A, Tsai YY, Sarcar B, Kahali S, Brem S, Yu HM, Rojiani A (2012). Phase I trial of vorinostat combined with bevacizumab and CPT-11 in recurrent glioblastoma. Neuro-Oncology.

[11] Friday BB, Anderson SK, Buckner J, Yu C, Giannini C, Geoffroy F, Schwerkoske J, Mazurczak M, Gross H, Pajon E (2012). Phase II trial of vorinostat in combination with bortezomib in recurrent glioblastoma: a north central cancer treatment group study. Neuro-Oncology.

[12] Galanis E, Anderson SK, Miller CR, Sarkaria JN, Jaeckle K, Buckner JC, Ligon KL, Ballman KV, Moore DF, Nebozhyn M (2018). Phase I/II trial of vorinostat combined with temozolomide and radiation therapy for newly diagnosed glioblastoma: results of Alliance N0874/ABTC 02. Neuro-Oncology.

[13] Milne JC, Lambert PD, Schenk S, Carney DP, Smith JJ, Gagne DJ, Jin L, Boss O, Perni RB, Vu CB (2007). Small molecule activators of SIRT1 as therapeutics for the treatment of type 2 diabetes. Nature.

[14] Pacholec M, Bleasdale JE, Chrunyk B, Cunningham D, Flynn D, Garofalo RS, Griffith D, Griffor M, Loulakis P, Pabst B (2010). SRT1720, SRT2183, SRT1460, and resveratrol are not direct activators of SIRT1. J Biol Chem.

[15] Huber JL, McBurney MW, Distefano PS, McDonagh T (2010). SIRT1-independent mechanisms of the putative sirtuin enzyme activators SRT1720 and SRT2183. Future Med Chem.

[16] Gu W, Roeder RG (1997). Activation of p53 sequence-specific DNA binding by acetylation of the p53 C-terminal domain. Cell.

[17] Scuto A, Kirschbaum M, Buettner R, Kujawski M, Cermak JM, Atadja P, Jove R (2013). SIRT1 activation enhances HDAC inhibition-mediated upregulation of GADD45G by repressing the binding of NF-kappaB/STAT3 complex to its promoter in malignant lymphoid cells. Cell Death Dis.

[18] Gurt I, Artsi H, Cohen-Kfir E, Hamdani G, Ben-Shalom G, Feinstein B, El-Haj M, Dresner-Pollak R (2015). The Sirt1 activators SRT2183 and SRT3025 inhibit RANKL-induced Osteoclastogenesis in bone marrow-derived macrophages and Down-regulate Sirt3 in Sirt1 null cells. PLoS One.

[19] Yorimitsu T, Klionsky DJ (2007). Endoplasmic reticulum stress: a new pathway to induce autophagy. Autophagy.

[20] Mathew R, Karantza-Wadsworth V, White E (2007). Role of autophagy in cancer. Nat Rev Cancer.

[21] Chen LF, Mu Y, Greene WC (2002). Acetylation of RelA at discrete sites regulates distinct nuclear functions of NF-kappaB. EMBO J.

[22] Yuan ZL, Guan YJ, Chatterjee D, Chin YE (2005). Stat3 dimerization regulated by reversible acetylation of a single lysine residue. Science.

